# Cost-Effectiveness Analysis of Bariatric Surgery and Pharmacological Treatments for People with Obesity and Type 2 Diabetes Mellitus

**DOI:** 10.1007/s11695-026-08639-9

**Published:** 2026-04-01

**Authors:** Dolunay Özlem Zeybek, Vahit Yiğit

**Affiliations:** 1 Department of Health Management, Faculty of Health Sciences, Bandırma Onyedi Eylül University, Balıkesir, Turkey; 2 Department of Health Management, Faculty of Economics and Administrative Sciences, Süleyman Demirel University, Isparta, Turkey

**Keywords:** Type 2 Diabetes, Bariatric Surgery, Sleeve Gastrectomy, Markov Model, Cost-Effectiveness Analysis, Türkiye

## Abstract

**Objectives:**

Obesity and its associated type 2 diabetes mellitus (T2DM) represent a growing public health challenge worldwide. This study aimed to evaluate the cost-effectiveness of sleeve gastrectomy (SG) compared with pharmacological therapy (PT) in patients with obesity and T2DM from the perspective of the reimbursement institution in Türkiye.

**Methods:**

A Markov cohort model was developed to evaluate the cost-effectiveness of SG compared with PT in patients with obesity and T2DM. The model simulated a cohort of 1,000 individuals entering the model at age 40 years over a 60-year lifetime horizon from the perspectives of the Social Security Institution (SGK) and the Public Health Service Price Schedule (PHSP). Cost data were obtained from hospital records of 44 patients receiving PT and 55 patients undergoing SG, while effectiveness inputs were derived from published literature. Costs were calculated for the 2023 price year, and both costs and health outcomes were discounted at an annual rate of 3%. One-way and probabilistic sensitivity analyses were performed to assess parameter uncertainty.

**Results:**

According to the Markov model results, lifetime total costs were estimated at US$3,412.9 for PT and US$4,043.8 for SG. Lifetime health outcomes, measured in quality-adjusted life years (QALYs), were 11.33 for PT and 12.68 for SG. Compared with PT, SG provided an additional 1.35 QALYs at an incremental cost of US$630.9, corresponding to an incremental cost-effectiveness ratio (ICER) of US$467 per QALY gained.

**Conclusions:**

The results indicate that SG is a cost-effective treatment option for patients with obesity and T2DM in Türkiye, providing greater health benefits at an acceptable incremental cost compared with PT. The relatively lower healthcare costs in Türkiye may further enhance the long-term cost-effectiveness of SG.

**Supplementary Information:**

The online version contains supplementary material available at 10.1007/s11695-026-08639-9.

## Key Points

• SG is cost-effective compared with PT in people with obesity and T2DM.

• SG provides additional quality-adjusted life years (QALYs) compared with PT.

• Lower healthcare service costs in Türkiye enhance the cost-effectiveness of SG.

**Supplementary Information:**

The online version contains supplementary material available at 10.1007/s11695-026-08639-9.

## Introduction

Obesity and diabetes, which have become global problems, are among the most important chronic diseases of our time and are often described as epidemics [1]. In Türkiye, 61% of the population is overweight and 32.1% has obesity, making it the country with the highest prevalence of obesity in Europe. Obesity is a chronic disease associated with multiple obesity-associated medical problems, one of the most important being type 2 diabetes mellitus (T2DM), and approximately 80–90% of people with T2DM have obesity [2, 3]. In Türkiye, 14.5% of the population has diabetes, placing the country among the top five in Europe in terms of prevalence [4] .

The high prevalence of obesity and diabetes in Türkiye imposes a significant economic burden on the national healthcare budget [5, 6]. The increasing prevalence of T2DM and its associated economic burden pose challenges for decision-makers in managing the disease. Economic evaluation studies in healthcare are utilized to allocate scarce resources efficiently and to provide decision-makers with evidence-based information [7, 8]. Therefore, evaluating alternative treatment options for these conditions to identify the most appropriate therapy is crucial for enabling decision-makers to make informed choices [9]. In this context, treatment strategies for obesity and T2DM should be evaluated not only in terms of clinical effectiveness but also with regard to public budget sustainability and resource allocation.

Alternative treatment approaches for people with obesity and T2DM include medical nutrition therapy, physical activity, cognitive behavioral therapy, pharmacological treatment, and bariatric surgery [10]. Although there are different methods of bariatric surgery, SG has been the most commonly performed procedure worldwide and in Türkiye in recent years [11, 12]. Identifying the most cost-effective option among these alternative treatments is important to guide decision-makers and to reduce the financial burden on the national economy. A review of the literature reveals that economic evaluation studies have examined the costs and effectiveness of bariatric surgery and other alternative treatments in the management of people with obesity and T2DM. However, no cost-effectiveness studies on this topic have been conducted in Türkiye [13, 14]. This study aims to provide evidence-based information for healthcare policy decision-makers regarding resource allocation by evaluating the cost-effectiveness of SG and PT in the management of people with obesity and T2DM in the context of Türkiye.

## Methods

### Model Structure and Key Assumptions

The Markov transition model consisted of three health states: T2DM, remission, and death. At the beginning of the simulation, all individuals entered the model in the T2DM state. During each cycle, individuals in the T2DM state could either remain in the same state or transition to the death state. Individuals in the remission state could remain in remission, transition back to the T2DM state, or transition to the death state. Death was considered an absorbing state; therefore, transitions to death were possible from all health states [15].

In the model, remission was defined as the absence of glucose-lowering PT with normal glycemic control, consistent with definitions commonly used in the literatüre [16, 17]. However, remission was assumed to occur only after two years of treatment. Therefore, patients were assumed to continue T2DM-related PT during the first two years before entering the remission state. After remission, antidiabetic medications were discontinued, while symptomatic treatments for obesity-associated medical problems were assumed to continue with reduced intensity.

Transition probabilities between states in the Markov model were obtained from cohort studies or derived from the literatüre[18]. To date, no cohort study has been conducted in Türkiye on the treatment of people with obesity and T2DM [15]. Within the defined time horizon, individuals could occupy only one health state at a time. Age-specific mortality rates for Türkiye obtained from national life tables were used to estimate transition probabilities to the death state in the model [19]. The model cycle length was set at one year, with a time horizon of 60 years (lifetime).

Studies using Markov models to evaluate the cost-effectiveness of bariatric surgery in people with obesity and T2DM have used varying time horizons, including five years, ten years, and lifetime horizons [20–24]. Overall, previous studies have generally employed a lifetime horizon. In this study, a lifetime horizon of 60 years was adopted. Individuals were assumed to enter the model at the age of 40 years, reflecting the typical age at which bariatric surgery is commonly performed among adults with obesity and T2DM in clinical practice. A maximum life expectancy of 100 years was assumed to ensure that all relevant long-term costs and health outcomes were captured within the model. This upper age limit represents a technical modeling assumption to reflect a lifetime horizon rather than an assumption that individuals would necessarily live to this age.

Although the mean ages of the SG and PT groups differed in the observed patient sample, a common starting age of 40 years was used in the Markov model to represent a hypothetical cohort of patients with obesity and T2DM. This approach is commonly used in health economic modeling to ensure comparability between treatment strategies and to simulate long-term outcomes over a lifetime horizon.

The analysis was conducted from the perspective of the Social Security Institution (SGK), the main public health insurance payer in Türkiye, and the Public Health Service Price Schedule (PHSP), which provides reference prices for healthcare services delivered in public hospitals. Although healthcare services in Türkiye may also be financed through private health insurance and out-of-pocket payments, the majority of healthcare expenditures are covered by SGK. Therefore, SGK represents the primary reimbursement perspective in the Turkish healthcare system. In addition, the PHSP provides reference prices for services delivered in public hospitals, including services provided to uninsured individuals and international patients. From these perspectives, only direct medical costs related to the management of obesity and T2DM were included in the analysis.

**Fig. 1:**
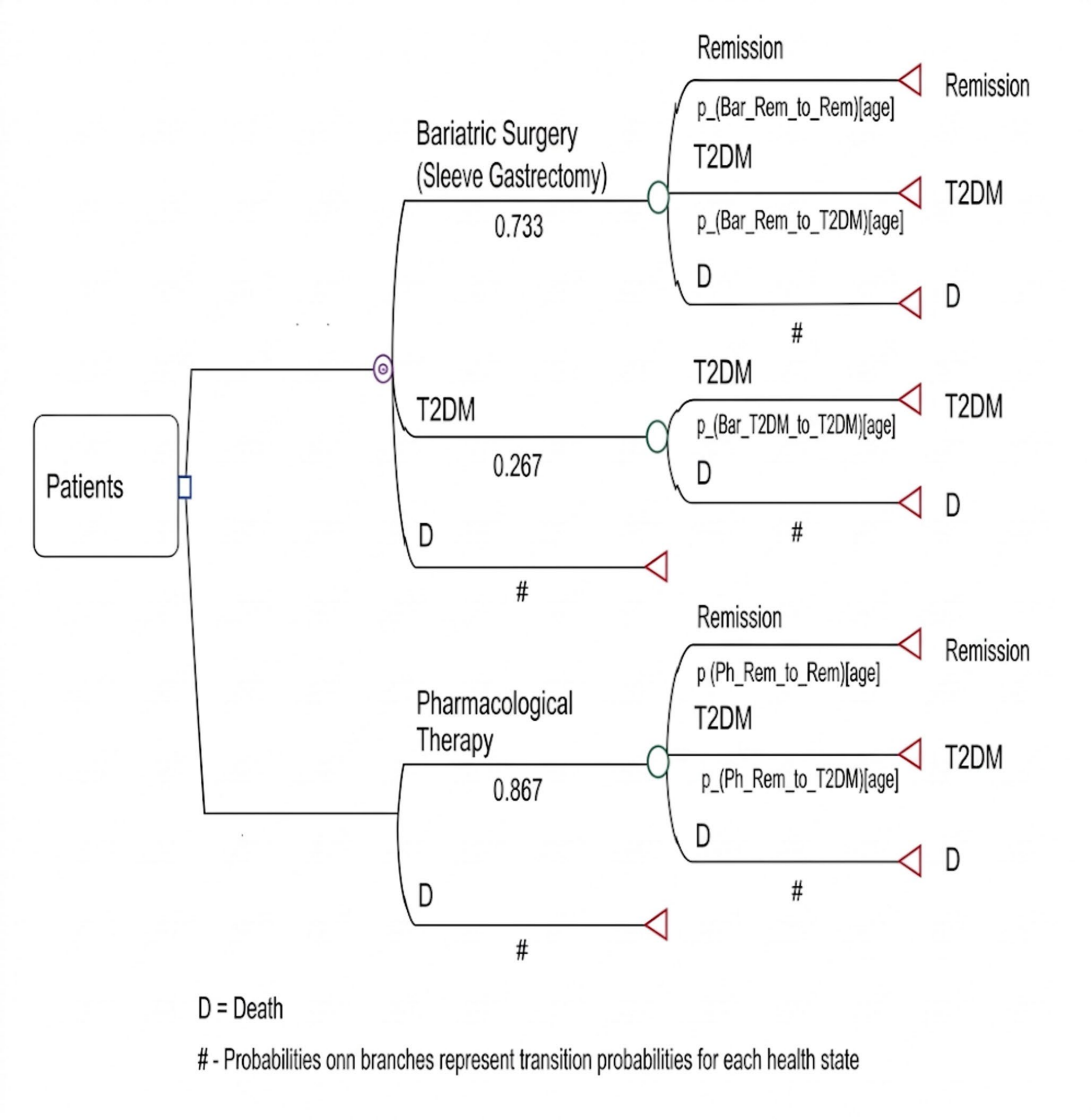
Markov model structure for patients with obesity and T2DM receiving SG or PT

A discount rate of 3% was applied in this study, reflecting Türkiye’s classification as an upper-middle-income country [25] and consistent with the rate commonly used in previous studies [23, 24, 26–30]. The discount rate was applied to both costs and health outcomes (QALYs). In the sensitivity analysis, a discount rate of 5% was applied. The model was developed using TreeAge Pro for Healthcare 2023 (TreeAge Software Inc., Williamstown, MA, USA). A Markov model was employed to evaluate the costs and effectiveness of SG and PT in the treatment of people with obesity and T2DM [15]. The structure of the Markov decision tree is presented in Fig. 1. The study was reported in accordance with the CHEERS reporting guidelines [31], and the completed CHEERS checklist is provided as supplementary material.

### Target Population

The study did not employ a sampling method. Instead, all patients with obesity and T2DM who visited two university hospitals between 01 January 2020 and 31 December 2021 were included if they were aged 18 years or older, had newly diagnosed T2DM (≤ 2 years), and had a BMI ≥ 35 kg/m². The target population of the study comprised 44 patients receiving PT and 55 patients who underwent SG and met these criteria. SG is a bariatric surgical procedure in which the greater curvature of the stomach is removed, resulting in the formation of a tubular gastric sleeve and reduced gastric capacity.

Patients in the PT group received routine medical management for obesity and T2DM in internal medicine outpatient clinics. The treatment primarily consisted of oral glucose-lowering agents such as metformin, sulfonylureas, SGLT2 inhibitors, and DPP-4 inhibitors, and no patients in the PT group received insulin therapy.

In the PT group, 42 patients were female and 2 were male, with a mean age of 50.1 years, whereas in the SG group, 40 patients were female and 15 were male, with a mean age of 35.8 years.

### Cost

Cost data for SG and PT were obtained from patient financial records in the hospital information system. Medication costs in the PT group were determined retrospectively based on patient prescription records. All prescribed medications were reviewed individually and grouped according to the Anatomical Therapeutic Chemical (ATC) classification system to reflect real-world PT patterns observed in the study cohort. Medication costs were calculated by weighting drug use according to the proportion of patients receiving each treatment in the cohort. The treatment primarily consisted of oral glucose-lowering agents such as metformin, sulfonylureas, SGLT2 inhibitors, and DPP-4 inhibitors. No patients in the study cohort received insulin therapy. In addition, glucose test strips used for home blood glucose monitoring were included in the cost calculations. Medications used for obesity treatment, as well as drugs prescribed for the symptomatic treatment of obesity-associated medical problems, were also incorporated into the cost analysis. For SG, cost items were calculated separately for the first and second years. In the first year, the cost items included consultation, tests and examinations, surgery, and medication. In the second year, the costs comprised outpatient visits and tests/examinations. For people with obesity and T2DM receiving outpatient care, annual cost data included consultation, tests and examinations, consultations with specialists, and medication costs. These costs were limited to healthcare utilization related to the management of obesity and T2DM and did not include all-cause healthcare utilization or the full costs of other obesity-associated medical problems. To calculate the costs, the Social Security Institution Health Implementation Communiqué (SUT) dated 19 October 2023 [32] and the PHSP dated 11 September 2023 [33] were used. All costs were standardized to the 2023 price year using these national price schedules and were expressed in US dollars (US$). Therefore, inflation and future medical cost growth were not modeled separately in the analysis. Costs were converted to US dollars using the exchange rate of the Central Bank of the Republic of Türkiye on 19 October 2023 (1 US$ = 28.0153 TRY) [34]. Table 1 presents the annual direct medical costs of therapeutic options for T2DM.

**Table 1:** Annual direct medical costs of therapeutic options in T2DM

Cost Item	SUT-Based Cost (US$)	PHSP-Based Cost (US$)
PT (Annual cost for Year 1 and Year 2)		
Outpatient Clinic (Per outpatient case fee – consultation, tests, and examinations)	39.19	53,54
Consultation	0.34	1.68
Tests and Examinations	7.20	10.58
Drug Costs		
T2DM	54.26	75.15
Obesity	556.45	772.85
Symptomatic	38.98	61.75
Medical Device Costs		
Strip	1.45	3.45
Total	**697.88**	**975.55**
PT (Annual cost for Year 3 and beyond)		
Outpatient Clinic (Per outpatient case fee – consultation, tests, and examinations)	26.13	35.69
Consultation	0.34	1.68
Tests and Examinations	7.20	10.58
Drug Costs		
T2DM	54.26	75.15
Symptomatic	38.98	65.77
Medical Device Costs		
Strip	1.45	3.45
Total	128.36	**192.32**
SG (Annual cost for Year 1)	H SUT-Based Cost (US$)	PHSP-Based Cost (US$)
Outpatient Clinic (Per outpatient case fee – consultation, tests, and examinations)	71.85	**98.16**
Operation	1,171.16	2,342.30
Consultation	0.34	1.68
Tests and Examinations	7.20	10.58
Drug Costs		
T2DM	54.26	75.15
SG	47.59	65.77
Symptomatic	38.98	61.75
Medical Device Costs		
Strip	1.45	3.45
Total	**1**,**391.40**	**2**,**658.87**
SG (Annual cost for Year 2 and beyond)	SUT-Based Cost (US$)	PHSP-Based Cost (US$)
Outpatient Clinic (Including consultation, tests, and examinations)	32.66	44.62
Consultation	0.34	1.68
Tests and Examinations	7.20	10.58
Drug Costs		
T2DM	54.26	75.15
Symptomatic	38.98	61.75
Medical Device		
Strip	1.45	3.45
Total	**133.45**	**197.23**

For people with T2DM, the annual treatment cost of PT from the third year onwards was calculated as 128.36 US$ based on SUT tariffs and 188.30 US$ based on PHSP tariffs. For SG, the annual treatment cost from the second year onwards was 133.45 US$ based on SUT tariffs and 197.23 US$ based on PHSP tariffs (Table 1). Table 2 presents the costs in remission based on both SUT and PHSP tariffs.

**Table 2:** Annual direct medical costs of therapeutic options in remission

Cost Item	SUT-Based Cost (US$)	PHSP-Based Cost (US$)
PT (Annual cost for Year 1 and Year 2)		
Outpatient Clinic (Per outpatient case fee – consultation, tests, and examinations)	39.19	53.54
Consultation	0.34	1.68
Dietitian	–	–
Obstetrics & Gynecology	–	–
Sports Medicine	–	–
Tests and Examinations	7.20	10.58
25-Hydroxy Vitamin D	–	–
Glycated Hemoglobin (HbA1c) (HPLC)	–	–
EMG, Polyneuropathy Protocol	–	–
Drug Costs		
T2DM	54.26	75.15
Obesity	556.45	772.85
Symptomatic Treatment	38.98	61.75
Medical Device		
Strip	1.45	3.45
Total	**697.88**	**975.55**
PT (Annual cost for Year 3 and beyond)	SUT-Based Cost (US$)	PHSP-Based Cost (US$)
Outpatient Clinic (Per outpatient case fee – consultation, tests, and examinations)	13.06	17.85
Glycated Hemoglobin (HbA1c) (HPLC)	6.54	9.07
Drug Costs		
Symptomatic Treatment	29.23	46.31
Total	**48.83**	**73.23**
SG (Annual cost for Year 1)	SUT-Based Cost (US$)	PHSP-Based Cost (US$)
Outpatient Clinic (Per outpatient case fee – consultation, tests, and examinations)	71.85	98.16
Operation	1,171.16	2,342.33
Consultation	0.34	1.68
Tests and Examinations	7.20	10.58
Drug Costs		
T2DM	54.26	75.15
SG	47.59	65.77
Symptomatic	38.98	61.75
Medical Device		
Strip	1.45	3.45
Total	**1**,**391.40**	**2**,**658.87**
SG (Annual cost for Year 2 and beyond)	SUT-Based Cost (US$)	PHSP-Based Cost (US$)
Outpatient Clinic (Per outpatient case fee – consultation, tests, and examinations)	19.60	26.77
Glycated Hemoglobin (HbA1c) (HPLC)	6.54	9.07
Drug Costs		
Symptomatic Treatment	29.23	46.31
Total	**55.37**	**82.15**

For both remission and T2DM states, the annual costs for the first and second years for PT, as well as the first-year costs for SG, were assumed to be the same. In the remission state, the annual treatment cost of PT from the third year onwards was estimated at 48.83 US$ using SUT tariffs and 73.23 US$ using PHSP tariffs. The annual treatment cost for SG from the second year onwards was 55.37 US$ using SUT tariffs and 82.15 US$ using PHSP tariffs.

### Measurement of Outcomes

In this study, alternative treatment strategies for people with obesity and T2DM were compared in terms of cost and effectiveness using a cost–utility analysis. Therefore, QALYs, which are commonly used in cost–utility analyses to measure health outcomes, were calculated. Due to the lack of institutional permission, effectiveness data could not be collected directly from patient records. Using effectiveness parameters from published literature is a widely accepted approach in economic evaluation studies.

Health-related quality-of-life utility values used for QALY estimation were obtained from the literature. The utility value for the T2DM health state was derived from the meta-analysis conducted by Redenz et al. [35], which reported a pooled utility value of 0.772 for patients with T2DM. Because Türkiye-specific utility values for patients in remission were not available, the remission utility value (0.812) was derived from the general population utility estimate reported by Falk Hvidberg and Hernández Alava [36].

The primary outcome of the economic evaluation was the ICER, calculated as the difference in total costs divided by the difference in QALYs between SG and PT. In addition, the NMB was calculated to further assess cost-effectiveness across different WTP thresholds.

### Threshold

ICER alone is not sufficient for reimbursement decisions. Therefore, in cost-effectiveness analyses, the ICER is compared with a threshold value to determine whether an intervention should be included in the reimbursement scheme [24, 25]. WTP threshold is typically expressed per QALY gained. The WHO recommends considering an intervention as cost-effective if the ICER per QALY gained is equal to or below three times the per capita GDP [26, 27]. Accordingly, in this study, the threshold was set at three times Türkiye’s 2023 per capita GDP, corresponding to 39,330 US$ [37].

### Sensitivity Analysis

To assess the impact of parameters that could influence the cost-effectiveness analysis results, best- and worst-case scenario sensitivity analyses were performed using all assumptions. One-way sensitivity analysis was conducted using a tornado diagram, evaluating the ICER and NMB of SG and PT in people with obesity and T2DM. In the probabilistic sensitivity analysis (PSA), study outcomes were examined using various WTP threshold values, and acceptability curves and scatter plots were generated on the cost-effectiveness plane. Additionally, Monte Carlo simulations were performed with 1,000 iterations and 500 samples, recalculating the incremental costs per QALY for alternative treatments and presenting the results in distribution graphs.

## Results

This study evaluated the cost-effectiveness of SG and PT in people with obesity and T2DM from the perspective of the SGK and using PHSP tariffs. Analyses were conducted using a 1,000-patient Markov cohort model with annual cycles over a 60-year (lifetime) time horizon. Outcomes were assessed in terms of total costs, QALYs, ICER, and NMB. The results of the study are presented in Table 3.

**Table 3:** Results of the cost-effectiveness analysis

Perspective	Treatment	Total Cost (US$)	Incremental Cost (US$)	Total QALYs	Incremental QALYs	Cost per QALY (US$)	ICER ((US$)/QALY))	Decision
SGK	PT	3.412,9	–	11,33	–	300,7	–	–
	SG	4.043,8	630,9	12,68	1.35	318,6	467	Cost-effective
PHSP	PT	4.869,4	–	11,33	–	429,4	–	–
	SG	7.157,9	2.288,5	12,68	1.35	564,3	1.697,6	Cost-effective

Over the 60-year time horizon, SG provided greater health gains compared to PT. The cumulative QALYs for SG treatment were 12.68, whereas they were 11.33 for PT. From the SGK perspective, the total costs for SG and PT were 4,043.8 US$ and 3,412.9 US$, respectively. Using PHSP tariffs, the corresponding costs were 7,157.9 US$ for SG and 4,869.4 US$ for PT.

The cost-effectiveness analysis showed that SG provided an additional 1.35 QALYs compared to PT. From the SGK perspective, the incremental cost of SG was 630.9 US$, corresponding to an ICER of 467 US$/QALY. Using PHSP tariffs, the incremental cost was 2,288.5 US$, with an ICER of 1,697.6 US$/QALY (Table 3). In both scenarios, the calculated ICER values were below the established willingness-to-pay threshold.

**Fig. 2:**
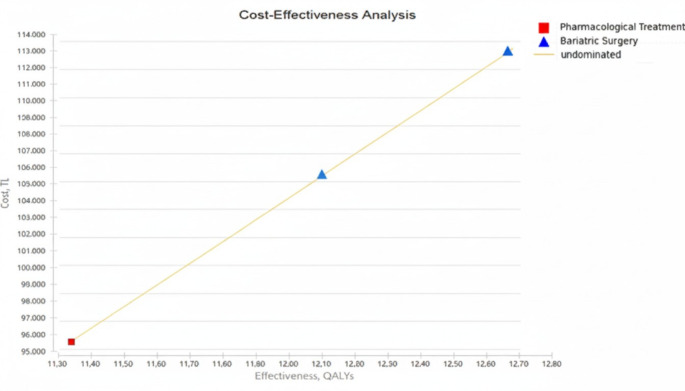
Distribution of PT and SG treatments on the cost-effectiveness plane

In the cost-effectiveness plane (Fig. 2), the Y-axis represents costs and the X-axis represents effectiveness measured in QALYs. The cost-effectiveness frontier is constructed according to the WTP threshold. Examination of the plane shows that SG is located in the first quadrant relative to PT. This indicates that SG is more effective and incurs higher costs compared to PT. In both perspectives, the ICER values obtained are below the WTP thresholds accepted for Türkiye: very cost-effective if below one times the per capita GDP (TRY307,952 ≈ US$10,994) and cost-effective if below three times the per capita GDP (TRY923,856 ≈ US$39,330). This demonstrates that SG represents a cost-effective treatment option compared to PT (Fig. 2).

In addition to ICER, the NMB analysis showed that SG treatment had a higher NMB compared to PT, and the incremental NMB was positive. These findings indicate that SG treatment remains economically advantageous even under different WTP threshold values. Overall, SG represents a cost-effective treatment option compared to PT in the treatment of people with obesity and T2DM across both perspectives.

**Fig. 3:**
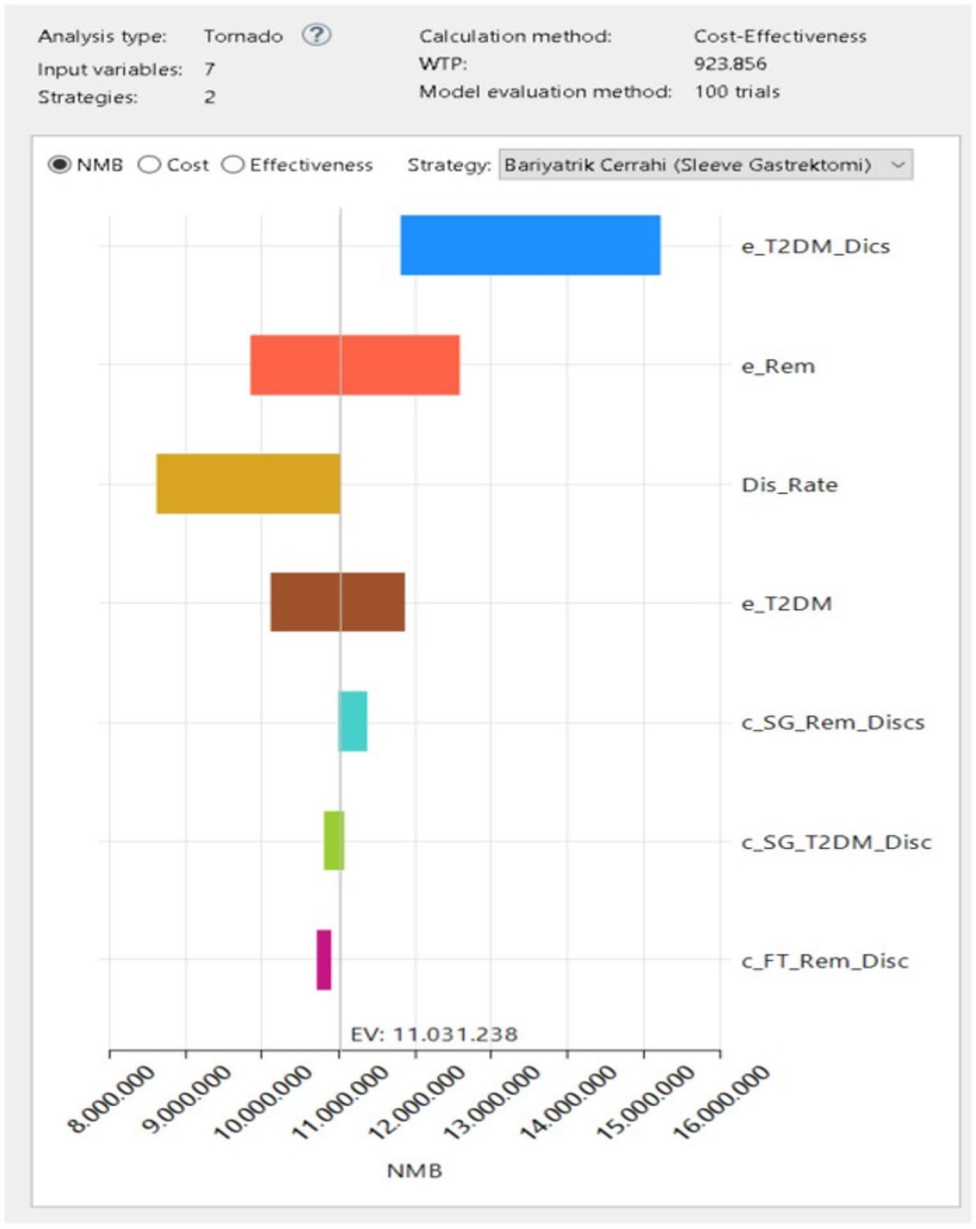
Tornado diagram showing the results of the one-way sensitivity analysis for the NMB of SG compared with PT

To assess uncertainty in the cost-effectiveness results, one-way sensitivity analysis and PSA were conducted. In the one-way sensitivity analysis, model parameters were varied across wide ranges, and evaluations were performed using the NMB criterion at the WTP threshold. Examination of the tornado diagram (Fig. 3) indicated that the parameters most affecting the cost-effectiveness of SG were the treatment effectiveness parameters for T2DM, the effectiveness parameters in the remission state, and the discount rate. Across all tested ranges, SG’s NMB values remained positive, and the decision outcome did not change.

PSA assessed uncertainties in model parameters using Beta and Gamma distributions. Each model parameter was run through a Monte Carlo simulation with 1,000 hypothetical iterations and 500 samples, based on randomly assigned values in the program. Examination of the PSA results indicated that SG has a higher probability of being cost-effective compared with PT across different WTP thresholds. Notably, at WTP thresholds of 7,140 US$ (200,000 TRY) and above, the probability that SG is cost-effective remained consistently high. Overall, the sensitivity analysis results confirm that the primary finding— that SG represents a cost-effective treatment option compared with PT in people with obesity and T2DM—remains robust.

## Discussion

This study evaluated the lifetime cost-effectiveness of SG compared with PT in people with obesity and T2DM in Türkiye. The results showed that SG provided an additional 1.35 QALYs at an extra cost of 630.9 US$ compared with PT. The ICER was calculated as 467.9 US$/QALY. Since this value is well below the WTP threshold based on the GDP per capita used in the study, SG represents a cost-effective treatment option. Despite its high upfront costs, SG represents an economically rational treatment option due to the long-term health gains it provides.

A review of the literature indicates that BS has been found to be a cost-effective or dominant treatment option compared to conventional therapy for people with obesity and T2DM in most countries [13, [14]. However, in the majority of these studies, BS has been considered as a whole [38–42]. In recent years, SG has emerged as one of the most commonly performed BS procedures globally and in Türkiye [11], yet the number of studies evaluating it as a separate treatment strategy remains limited [43–45].

Studies treating SG as an independent strategy have been conducted in China [43], Australia [44], Hungary [45], and the United States [46]. In a study conducted in China, SG was evaluated in people with obesity and T2DM using a Markov simulation model; the cost per QALY of SG was calculated as 1,028.97 US$, and it was found to be a cost-effective option for T2DM treatment [43]. In a study from Australia, conducted from a health system perspective, the cost per QALY of SG was calculated as 27,523 US$, and was shown to be a cost-effective option compared to standard therapy [44]. In Hungary, a microsimulation model from a third-party payer perspective calculated the cost per QALY of SG as €17,064, confirming it as a cost-effective treatment option [45]. In the United States, Lauren et al.[47] calculated the cost per QALY of SG and demonstrated it as a cost-effective option compared to standard therapy. In the present study, SG provided an additional 1.35 QALYs compared to PT at an incremental cost of TRY17,666 (630.9 US$,), with an ICER of TRY13,108/QALY (467 US$/QALY). These findings are consistent with international studies from China, Australia, Hungary, and the United States, which have reported SG as cost-effective. Notably, the cost per QALY calculated in the current study is considerably lower than in other countries, indicating that SG is also an economically sustainable option in Türkiye.

The ICER values reported in international studies are monetarily higher than those observed in the present study. Studies conducted in the United States and Europe report ICERs for SG or BS ranging from €8,000 to €40,000 per QALY [30, 47, 48]. This discrepancy may be attributed to lower healthcare service prices in Türkiye, the lower exchange value of the Turkish lira, pharmaceutical pricing policies, and the fact that this analysis was conducted from the SGK perspective [49–51].

Other model-based studies that considered people with obesity and T2DM as a sub-cohort have also shown that BS is cost-effective in nearly all countries [28, 29, 40–42, 52]. The presence of T2DM in addition to obesity in patients has been shown to enhance the effectiveness gains of BS. Therefore, the additional QALY gain provided by SG compared with PT in this study is consistent with the results reported for T2DM sub-cohorts in the literature.

Overall, based on the literature and the results of this study, SG, one of the BS methods, represents good value for money compared with PT in people with obesity and T2DM. However, despite being a cost-effective treatment option for people with T2DM, SG continues to be associated with ethical debates. The ethical concerns related to BS include high costs, inequities and fairness in access to treatment, risk–benefit balance, insufficient clinical evidence, effectiveness and safety issues, patient autonomy, informed consent, healthcare professionals’ biases, stigmatization, and public perception. Although SG has been highlighted as cost-effective, issues such as “ethical concerns” and “inequities in access to treatment” have been emphasized. A key gap in the study’s implications is the lack of a strategy to manage the first-year budget impact on public finances. The high initial cost of SG may pose a barrier for decision-makers with short-term budget constraints; therefore, bridging cost-effectiveness with a budget impact analysis can be proposed as a critical recommendation [46, 53–57]. In conclusion, SG is not a universal solution for all patients. Ethical considerations, patient selection, surgical risks, and the need for long-term follow-up should be carefully addressed in health policy decisions.

Obesity imposes a substantial economic burden on healthcare systems in many countries. For example, a study conducted in the United States from a third-party payer perspective estimated the lifetime cost of obesity at approximately $171,482 per patient [58]. In addition, the direct healthcare costs of severe obesity in the United States were estimated to reach $69 billion in 2014 [59]. Similarly, studies conducted in Europe have shown that obesity represents a significant burden on healthcare systems. For instance, in Germany, the total economic cost of obesity has been estimated at €63.04 billion [60, 61]. Compared with these estimates, the lifetime healthcare costs calculated in the present study are considerably lower than those reported in the United States and European countries. This difference may be explained by relatively lower healthcare service prices in Türkiye, pharmaceutical pricing policies, and the payer perspective adopted in the present study. Consequently, the ICER values estimated in this study should be interpreted within the context of Türkiye’s healthcare cost structure and may not be directly comparable with results from countries where healthcare costs are substantially higher.

### Limitations

This study has several limitations. First, the number of patients included in the analysis was limited due to difficulties in accessing healthcare services during the COVID-19 pandemic. Data for individuals receiving SG and PT were obtained from hospital information systems, and these records were assumed to be accurate and complete.

Second, some parameters related to long-term complications, treatment effectiveness, and transition probabilities were obtained from the literature because Türkiye-specific longitudinal data were not available. Therefore, these effectiveness estimates were assumed to be applicable to individuals with obesity and type 2 diabetes mellitus in the study population.

Third, the model included only SG and PT, and other bariatric procedures or alternative treatment strategies were not evaluated. In addition, the analysis focused on individuals undergoing SG and those receiving oral antidiabetic therapy, and costs were calculated from the perspective of university hospitals.

Another limitation relates to the utility values used in the model. Because remission-specific utility values for individuals with type 2 diabetes mellitus were not available, general population utility estimates were used to represent the remission state. This assumption may overestimate the quality-of-life gains associated with remission.

Finally, healthcare costs in Türkiye are considerably lower than those reported in many Western healthcare systems. Therefore, the lifetime cost estimates and ICER values observed in this study may not be directly generalizable to countries with substantially higher healthcare expenditures.

## Conclusions

This study evaluated the cost-effectiveness of SG compared with PT for people with obesity and T2DM in Türkiye over a 60-year (lifetime) horizon using a Markov cohort model from the perspective of the SGK and based on PHSP tariffs. SG provided an additional 1.35 QALYs per patient compared with PT. The incremental cost of SG remained below the predefined WTP thresholds. One-way and probabilistic sensitivity analyses confirmed the robustness of the results. Overall, SG appears to be a cost-effective treatment option for people with obesity and T2DM in Türkiye and may provide useful evidence for healthcare decision-makers. However, the findings are subject to model assumptions and literature-based inputs, highlighting the need for Türkiye-specific long-term clinical and economic data.

## Supplementary Information

Below is the link to the electronic supplementary material.

Supplementary Material 1.

## Data Availability

No datasets were generated or analysed during the current study.

## References

[1] Barnes AS, Coulter SA. The Epidemic of Obesity and Diabetes: Trends and Treatments. Tex Heart Inst J. 2011;38:142. 21494521 PMC3066828

[2] Nianogo RA, Arah OA. Forecasting obesity and type 2 diabetes incidence and burden: The vila-obesity simulation model. Front Public Health. 2022;10:1–13. 10.3389/fpubh.2022.818816 10.3389/fpubh.2022.818816 PMC9016163 35450123

[3] WHO & IDF. Diagnosis and Management of Type 2 Diabetes. (2020) 10.1016/S0212-6567(10)70002-0

[4] International Diabetes Federation. IDF Diabetes Atlas. Int Diabetes Federation. 2021. 10.1016/j.diabres.2013.10.013. 35914061

[5] Yiğit A, Yiğit V. Economic Burden of Obesity-Related Comorbidities in Türkiye. Gümüşhane Üniversitesi Sağlık Bilimleri Dergisi Araştırma Makalesi GUJHS. 2019;8:223–30.

[6] Öcal EE, Önsüz MF. Diyabet Hastalığının Ekonomik Yükü. Türk Dünyası Uygulama ve Araştırma Merkezi Halk Sağlığı Dergisi. 2018;3:24–31.

[7] Chisholm D, Evans DB. Economic Evaluation in Health: Saving Money or İmproving Care. J Med Econ. 2007;10:325–37.

[8] Botchkarev A. Essential Notion of the Health Economic Evaluation: Definition. Atl Rev Econ. 2016;2:1–15.

[9] Drummond M. Methods for the Economic Evaluation of Health Care Programmes. J Epidemiol Community Health (1978). 2006;60:822. 3rd ed.

[10] TEMD. Obezite Tanı ve Tedavi Kılavuzu. Türkiye Endokrinoloji ve Metabolizma Derneği. 2017.

[11] Welbourn R, et al. Bariatric Surgery Worldwide: Baseline Demographic Description and One-Year Outcomes from the Fourth IFSO Global Registry Report 2018. Obes Surg. 2019;29:782–95. 30421326 10.1007/s11695-018-3593-1

[12] Özatkan Y. Obezite Cerrahisinin Yaşam Kalitesine Etkisi ve Maliyet: Hasta Perspektifinden Bir Değerlendirme. Ankara Üniversitesi; 2021.

[13] Jordan K, Fawsitt CG, Carty PG, Clyne B, Teljeur C, Harrington P, Ryan M. Cost-effectiveness of metabolic surgery for the treatment of type 2 diabetes and obesity: a systematic review of economic evaluations. Eur J Health Econ. 2023;24(4):575–590. 10.1007/s10198-022-01494-2 PMC10175448 35869383

[14] Zeybek DÖ, Yiğit V. Cost Effectiveness of Bariatric Surgical Treatment Methods: A Systematic Review. Anemon Muş Alparslan Üniversitesi Sosyal Bilimler Dergisi. 2023;11:1001–17.

[15] Keating CL, et al. Cost-Effectiveness of Surgically Induced Weight Loss for the Management of Type 2 Diabetes: Modeled Lifetime Analysis. Diabetes Care. 2009;32:567. 19171720 10.2337/dc08-1749 PMC2660478

[16] Kelly J, Karlsen M, Steinke G. Type 2 Diabetes Remission and Lifestyle Medicine: A Position Statement From the American College of Lifestyle Medicine. Am J Lifestyle Med. 2020;14:406–19. 33281521 10.1177/1559827620930962 PMC7692017

[17] Buse JB, et al. How Do We Define Cure of Diabetes? Diabetes Care. 2009;32:2133. 19875608 10.2337/dc09-9036 PMC2768219

[18] Alarid-Escudero F, et al. A Tutorial on Time-Dependent Cohort State-Transition Models in R using a Cost-Effectiveness Analysis Example. Med Decis Mak. 2023;43:21. 10.1177/0272989X221121747 PMC9844995 36112849

[19] TÜİK. Ölüm ve Ölüm Nedeni İstatistikleri. 2022. https://data.tuik.gov.tr/Bulten/Index?p=Olum-ve-Olum-Nedeni-Istatistikleri-2022-49679 (2023).

[20] Ackroyd R, Mouiel J, Chevallier JM, Daoud F. Cost-Effectiveness and Budget İmpact of Obesity Surgery in Patients With Type-2 Diabetes in Three European Countries. Obes Surg. 2006;16:1488–503. 17132416 10.1381/096089206778870067

[21] Anselmino M, et al. Cost-Effectiveness and Budget Impact of Obesity Surgery in Patients with Type 2 Diabetes in Three European Countries(II). Obes Surg. 2009;19:1542–9. 19756896 10.1007/s11695-009-9946-z

[22] Gil-Rojas Y, et al. Cost-Effectiveness of Bariatric Surgery Compared With Nonsurgical Treatment in People With Obesity and Comorbidity in Colombia. Value Health Reg Issues. 2019;20:79–85. 31082638 10.1016/j.vhri.2019.01.010

[23] McGlone ER et al. Bariatric Surgery for Patients with Type 2 Diabetes Mellitus Requiring İnsulin: Clinical Outcome and Cost-Effectiveness Analyses. PLoS Med 2020;17:1–22. 10.1371/journal.pmed.1003228 10.1371/journal.pmed.1003228 PMC7721482 33285553

[24] Assumpção RP, et al. Cost-Utility of Gastric Bypass Surgery Compared to Clinical Treatment for Severely Obese With and Without Diabetes in the Perspective of the Brazilian Public Health System. Obes Surg. 2019;29:3202–11. 31214966 10.1007/s11695-019-03957-7

[25] Rognoni C, Armeni P, Tarricone R, Donin G. Cost–Benefit Analysis in Health Care: The Case of Bariatric Surgery Compared With Diet. Clin Ther. 2020;42:60–e757. 31959413 10.1016/j.clinthera.2019.12.001

[26] Viratanapanu I et al. Cost-effectiveness evaluation of bariatric surgery for morbidly obese patients with diabetes in Thailand. J Obes. 2019:5383478:1–6. 10.1155/2019/5383478 10.1155/2019/5383478 PMC6377984 30863633

[27] World Bank. 2022. https://data.worldbank.org/?locations=XT-TR.

[28] Borisenko O, Lukyanov V, Ahmed AR. Cost–Utility Analysis of Bariatric Surgery. Br J Surg. 2018;105:1328–37. 29667178 10.1002/bjs.10857

[29] Borisenko O, Mann O, Duprée A. Cost-Utility analysis of bariatric surgery compared with conventional medical management in germany: A Decision Analytic Modeling. BMC Surg. 2017;17:1–9. 10.1186/s12893-017-0284-0 10.1186/s12893-017-0284-0 PMC5543597 28774333

[30] Hoerger TJ, et al. Cost-Effectiveness of Bariatric Surgery for Severely Obese Adults with Diabetes. Diabetes Care. 2010;33:1933–9. 20805271 10.2337/dc10-0554 PMC2928336

[31] Saygın Avşar T et al. Konsolide Sağlık Ekonomisi Değerlendirme Raporlama Standartları Türkçe: Sağlık Hizmetlerinin Ekonomik Değerlendirmesinde Raporlama Standartlarının Türkiye Uyarlaması. Türkiye Klinikleri Sağlık Bilimleri Dergisi. 2023;8(3):1–17. 10.5336/healthsci.2023-96248

[32] Republic of türkiye social security institution. Health implementation communiqué (SUT). Official Gazette: 24 March 2023; No: 32142.

[33] Ministry of Health (Republic of Türkiye). Public Healthcare Service Price Tariff. 2023. Sağlık Bakanlığı. Kamu Sağlık Hizmeti Fiyat Tarifesi. https://shgmsgudb.saglik.gov.tr/TR-99401/kamu-saglik-hizmetleri-fiyat-tarifesi-2023-yili.html

[34] TCMB. TCMB Kurlar Sayfası. 2023. Central Bank of the Republic of Türkiye (CBRT). Exchange Rates. 2023. https://www.tcmb.gov.tr/kurlar/202310/19102023.xml

[35] Redenz G, Ibaceta MC, Aceituno D, Balmaceda C, Espinoza MA. Health State Utility Values of Type 2 Diabetes Mellitus and Related Complications: A Systematic Review and Meta-Analysis. Value Health Reg Issues. 2023;34:14–22. 36371899 10.1016/j.vhri.2022.09.005

[36] Falk Hvidberg M, Hernández Alava M. Catalogues of EQ-5D-3L Health-Related Quality of Life Scores for 199 Chronic Conditions and Health Risks for Use in the UK and the USA. PharmacoEconomics. 2023;41:1287–388. 37330973 10.1007/s40273-023-01285-4 PMC10492737

[37] Turkish Statistical Institute. Gross Domestic Product. 2022. (2023). https://data.tuik.gov.tr/Bulten/Index?p=Yillik-Gayrisafi-Yurt-Ici-Hasila-2022-49742

[38] Gulliford MC, et al. Costs and Outcomes of Increasing Access to Bariatric Surgery: Cohort Study and Cost-Effectiveness Analysis Using Electronic Health Records. Value Health. 2017;20:85–92. 28212974 10.1016/j.jval.2016.08.734 PMC5338873

[39] Lucchese M, et al. Cost-Utility Analysis of Bariatric Surgery in Italy: Results of Decision-Analytic Modelling. Obes Facts. 2017;10:261–72. 28601866 10.1159/000475842 PMC5644931

[40] Borisenko O, Lukyanov V, Debergh I, Dillemans B. Cost-Effectiveness Analysis of Bariatric Surgery for Morbid Obesity in Belgium. J Med Econ. 2018;21:365–73. 29271279 10.1080/13696998.2017.1419958

[41] Borisenko O, Lukyanov V, Johnsen SP, Funch-Jensen P. Cost analysis of bariatric surgery in Denmark made with a decision-analytic model. Dan Med J. 2017;64(8):A5401. https://pubmed.ncbi.nlm.nih.gov/28869031/28869031

[42] Borisenko O, et al. Bariatric Surgery can Lead to Net Cost Savings to Health Care Systems: Results from a Comprehensive European Decision Analytic Model. Obes Surg. 2015;25:1559–68. 25639648 10.1007/s11695-014-1567-5 PMC4522026

[43] Tang Q et al. Cost-Effectiveness of Bariatric Surgery for Type 2 Diabetes Mellitus: A Randomized Controlled Trial in China. Medicine*. *2016;95(20):e3522. 10.1097/MD.0000000000003522 10.1097/MD.0000000000003522 PMC4902396 27196454

[44] James R, Salton RI, Byrnes JM, Scuffham PA. Cost-Utility Analysis for Bariatric Surgery Compared with Usual Care for the Treatment of Obesity in Australia. Surg Obes Relat Dis. 2017;13:2012–20. 28237564 10.1016/j.soard.2016.12.016

[45] Kovács G, Mohos E, Kis JT, Tabák Á, Gerendy P, Pettkó J, Nagy D, Győrbíró D, Kaló Z. Cost-Effectiveness of Bariatric Surgery in Patients Living with Obesity and Type 2 Diabetes. J Diabetes Res. 2023;2023:9686729:1–8. 10.1155/2023/9686729 10.1155/2023/9686729 PMC10748723 38144444

[46] Zeybek DÖ, Yiğit V. Bariyatrik Cerrahinin Etik Sorunları: Sistematik Bir İnceleme. SDÜ Sağlık Yönetimi Dergisi. 2024;6:167–80.

[47] Lauren BN, et al. Estimated Cost-effectiveness of Medical Therapy, Sleeve Gastrectomy, and Gastric Bypass in Patients with Severe Obesity and Type 2 Diabetes. JAMA Netw Open. 2021;E2148317. 10.1001/jamanetworkopen.2021.48317. 10.1001/jamanetworkopen.2021.48317 PMC8845022 35157054

[48] Ikramuddin S, Klingman CD, Swan T, Minshall ME. Cost-Effectiveness of Roux-en-Y Gastric Bypass in Type 2 Diabetes Patients. Am J Managed Care. 2009;15:607–15. 19747025

[49] Akbolat M, Deniz NG. Türkiye’de Medikal Turizmin Gelişimi Ve Bazı Ülkelerle Karşılaştırılması. Int J Global Tourism Res. 2017;1:123–39. https://dergipark.org.tr/en/pub/ijgtr/issue/33240/366061. Preprint at.

[50] Gürleyen B, Çınar F. Türkiye’nin Medikal Turizm SWOT Analizi: COVID-19 Örneği. Sağlık ve Sosyal Refah Araştırmaları Dergisi. 2021;3:51–60.

[51] IQVIA. Türkiye Pharmaceutical Sector Report. 2023. https://www.aifd.org.tr/wp-content/uploads/2023/12/IQVIA_TURKIYE-ILAC-SEKTORU_RAPORU_.pdf

[52] Sanchez-Santos R, et al. Bariatric Surgery Versus Conservative Management for Morbidly Obese Patients in Spain: A Cost-Effectiveness Analysis. Expert Rev Pharmacoecon Outcomes Res. 2018;18:305–14. 29188745 10.1080/14737167.2018.1407649

[53] Craig H, le Roux C, Keogh F, Finucane FM. How Ethical Is Our Current Delivery of Care to Patients with Severe and Complicated Obesity? Obes Surg. 2018;28:2078–82. 29766353 10.1007/s11695-018-3301-1 PMC6018590

[54] Dixon J, Obesity. Health economics of bariatric surgery-benefit versus cost. Nat Rev Endocrinol. 2012;8:632–3. 22965168 10.1038/nrendo.2012.172

[55] Hofmann B. Stuck in the middle: The many moral challenges with bariatric surgery. Am J Bioeth. 2010;10:3–11. 21161829 10.1080/15265161.2010.528509

[56] Puia A, Puia IC, Cristea PG. Ethical considerations in bariatric surgery in a developing country. Clujul Med. 2017;90:268–72. 28781522 10.15386/cjmed-733 PMC5536205

[57] Saarni SI, et al. Ethical issues of obesity surgery–a health technology assessment. Obes Surg. 2011;21:1469–76. 21479827 10.1007/s11695-011-0386-1

[58] Yang Z, Zhang N. The Burden of Overweight and Obesity on Long-Term Care and Medicaid Financing. Med Care. 2014;52(7):658–63. 24926714 10.1097/MLR.0000000000000154

[59] Wang YC, Pamplin J, Long MW, Ward ZJ, Gortmaker SL, Andreyeva T. Severe Obesity in Adults Cost State Medicaid Programs Nearly $8 Billion n 2013. Health Aff. 2015;34(11):1923–31. 10.1377/hlthaff.2015.0633 10.1377/hlthaff.2015.0633 26526251

[60] Okunogbe A, Nugent R, Spencer G, Ralston J, Wilding J. Economic Impacts of Overweight and Obesity: Current and Future Estimates for Eight Countries. BMJ Global Health. 2021;6:6351. 10.1136/bmjgh-2021-006351 PMC8487190 34737167

[61] Effertz T, Engel S, Verheyen F, Linder R. The Costs and Consequences of Obesity in Germany: A New Approach From a Prevalence and Life-Cycle Perspective. Eur J Health Economics: HEPAC : Health Econ Prev Care. 2016;17(9):1141–58. 10.1007/s10198-015-0751-4 26701837

